# Proteins associated with incident metabolic syndrome in population-based cohorts

**DOI:** 10.1186/s13098-021-00752-2

**Published:** 2021-11-10

**Authors:** Lars Lind, Johan Sundström, Johan Ärnlöv

**Affiliations:** 1 Department of Medical Sciences, Uppsala University, Uppsala, Sweden; 2 The George Institute for Global Health, University of New South Wales, Sydney, Australia; 3 School of Health and Social Sciences, Dalarna University, Falun, Sweden; 4 The Division of Family Medicine and Primary Care, Department of Neurobiology, Care Sciences and Society, Karolinska Institutet, Huddinge, Sweden

**Keywords:** Metabolic syndrome, Protein, Interleukin, Epidemiology, Prospective

## Abstract

**Background:**

The metabolic syndrome (MetS) identifies persons with clustering of multiple cardiometabolic risk factors. The underlying pathology inducing this clustering is not fully known. We used a targeted proteomics assay to identify associations of circulating proteins with MetS and its components, cross-sectionally and longitudinally.

**Methods:**

We explored and validated associations of 86 cardiovascular proteins, assessed using a proximity extension assay, with the MetS in two independent cohorts; the Prospective Investigation of the Vasculature in Uppsala Seniors (PIVUS, n = 996) and Uppsala Longitudinal Study of Adult Men (ULSAM, n = 785). The analyses were adjusted for smoking, exercise habits, education, and energy and alcohol intake.

**Results:**

Nine proteins were associated with all five components of the MetS in PIVUS using FDR < 0.05 in a cross-sectional analysis. Of those nine proteins, only Interleukin-1 receptor antagonist protein (IL-1RA) was associated with all five components of the MetS in ULSAM using p < 0.05. IL-1RA levels were associated with incident MetS (n = 109) in PIVUS during a 5-year follow-up (HR 1.76 for a 1 SD change (95% CI 1.38, 2.24), p = 4.3*10^–6^). IL-1RA was however not causally related to MetS in a two-sample Mendelian randomization analysis using published data.

**Conclusion:**

Circulating IL-1RA was related to all five components of the MetS in a cross-sectional analysis in two independent samples, as well as to incident MetS in a longitudinal analysis. However, Mendelian randomization analyses did not provide support for a causal role for IL-1RA in the development of MetS.

**Supplementary Information:**

The online version contains supplementary material available at 10.1186/s13098-021-00752-2.

## Background

The metabolic syndrome (MetS) is describing the clustering of multiple cardiovascular risk factors in the same individual [1–3]. Originally, insulin resistance was pointed out as the connecting link initiating this clustering [3], but also other pathophysiological links, such as visceral obesity, a reduced blood flow, inflammation, liver steatosis, genetic factors, and an increased sympathetic tone, have all been suggested to be of importance [4–11].

We have recently published data on a number of proteins that were related to all of the five different components of the MetS (consensus-NCEP criteria; glucose, blood pressure, triglycerides, HDL, and waist circumference) in a cross-sectional analysis in the EpiHealth cohort [12], in an attempt to discover possible molecular pathways involved in MetS development. In the present study, we used data from two other cohort studies in which we have measured multiple proteins by the proximity extension assay (PEA) [13] to test the hypothesis that proteins being linked to all five MetS components would also be associated with incident MetS and possibly causally involved in the development of MetS.

To test this hypothesis, we first used a cross-sectional discovery/replication-approach in two independent community-based samples investigating associations between circulating proteins and the five components of MetS. We therefore evaluated if proteins consistently being related to all five of the MetS components in both cohorts also were related to incident MetS during 5 years of follow-up in the Prospective Investigation of the Vasculature in Uppsala Seniors (PIVUS) study. As a third step, we evaluated if proteins being associated with incident MetS also were causally related to MetS using Mendelian randomization analysis.

## Methods

### Study population

The Prospective Investigation of the Vasculature in Uppsala Seniors (PIVUS) study consists of 996 subjects with complete data who, at the age of 70 years, were examined regarding cardiovascular risk factors and vascular function [14] in 2001–2004. This cohort was reinvestigated 5 years later (n = 826).

The Uppsala Longitudinal Study of Adult Men (ULSAM) study consists of 2232 men who at the age of 50 were examined regarding cardiovascular risk factors [15] in 1970–1974. This cohort has since then been reinvestigated multiple times and the present study used data from the reinvestigation at age 77 years (in 1997–2001) when the same proteins as evaluated in PIVUS were measured (n = 785).

Baseline characteristics for the two cohorts can be found in Table 1.

**Table 1:** Basic characteristics and life-style factors in the ULSAM and PIVUS studies. Mean (SD) or proportions are given. MetS = metabolic syndrome

	PIVUS	ULSAM	p-value
n	996	785	
Age (years)	70 (0.1)	77 (0.8)	< 0.001
Sex (% female)	50	0	–
Energy intake (kcal)	1881 (468)	–	–
Alcohol intake (g/day)	6.3 (7.0)	–	–
Education	1–9 years: 57%	1–9 years: 63%	< 0.001
	10–12 years: 18%	10–12 years: 27%	
	> 12 years: 25%	> 12 years: 10%	
Current smokers (%)	11	2.4	0.024
Exercise habits	Sedentary:11%	Sedentary: 8%	< 0.001
	Light: 59%	Light: 35%	
	Regular: 23%	Regular: 53%	
	Athlete: 7%	Athlete: 4%	
MetS (%)	23	27	0.090
Glucose criteria (%)	21	24	0.23
HDL criteria (%)	17	19	0.42
Triglyceride criteria (%)	17	25	< 0.001
Blood pressure criteria (%)	83	90	< 0.001
Waist circumference criteria (%)	35	22	< 0.001

### Proteomics

Ninety-two proteins were measured in plasma by the proximity extension assay (PEA) [13]. The proteins analyzed in the present study were preselected to have a relationship with cardiovascular disease (the CVD chip 1, OLINK, Uppsala, Sweden) [16]. 86 of the proteins showed a call-rate > 75% and were used in the analysis. All but 8 showed a call-rate > 95% and 67 showed a call-rate of 100%. Values below the level of detection (LOD) were imputed as LOD/2^0.5. The imputed datasets were used in the further analyses of the proteins. The protein levels were log2-transformed to achieve normal distributions. This was checked by inspection of histograms. The values were further transformed to a standard deviation (SD)-scale (also called Z-scale) to produce comparable beta-estimates in the downstream analyses. The protein analyses in the two population-samples were performed by the same laboratory on frozen plasma samples (-80℃) drawn in the fasting state during the fall of 2014.

### Traditional risk factors

Blood was drawn in the morning after an overnight fast. Fasting HDL- and LDL-cholesterol and glucose were measured at the department of Clinical Chemistry at the University Hospital, Uppsala, by standard techniques. Blood pressure was measured in the supine position after 5–15 min of rest with a mercury sphygmomanometer. Waist circumference was measured av the umbilical level. BMI was calculated as weight divided by squared height. The measurements were performed in the same manner in the two cohorts. See [14, 15] for details.

### Life-style factors

All life-style factors were self-reported. Exercise habits were given on a 4 level scale based on two questions; how many times a week do you engage in regular exercise for at least 30 min which does not make you sweat/which makes you sweat? The lowest level was defined as light exercise < 2 times a week and no heavy exercise. Next level as light exercise > 1 times a week and no heavy exercise. Next level as heavy exercise 1–2 times a week and highest level as heavy exercise > 2 times a week. Smoking was defined as current smoking. Alcohol was determined from questions on different kind of alcohol and calculated as g/week. Energy intake was given from a 7-day dietary record and calculated from a variety of food items (given in kcal/day). The subjects were asked how many years they have been in school/university.

### Metabolic syndrome

The MetS was defined according to the consensus NECP (National Cholesterol Education Program) criteria [1] and the five components were defined as follows: Blood pressure ≥ 130/85 mmHg or antihypertensive treatment, fasting plasma glucose ≥ 6.1 mmol/l or antidiabetic treatment, serum triglycerides ≥ 1.7 mmol/l, waist circumference > 102 cm in men and > 88 cm in women, HDL-cholesterol < 1.0 mmol/l in men and < 1.3 in women. Three of the mentioned five criteria should be fulfilled for MetS.

### Statistical methods

In the descriptive Table 1, one-way factorial ANOVA was used for the continuous variables and the chi-square test for the nominal variables to test for differences between the two samples.

The PIVUS sample was used as the discovery dataset and ULSAM as replication in the cross-sectional analysis. In this analysis, a logistic regression analysis with one of the components of MetS was carried out for each of the 86 proteins. Thus, 86*5 models were analyzed.

In the discovery analysis, these models were adjusted for sex (age same in all subjects in PIVUS) and the life-style factors smoking, alcohol and energy intake, exercise habits, and education level, as well as storage time in the freezer. In the validation step (in ULSAM), the models were adjusted for age and the life-style factors smoking, exercise habits, and education level (alcohol and energy intake not known), as well as storage time in the freezer. Results with a false discovery rate (FDR) < 0.05 in the discovery step were further evaluated in the validation step and at this stage a nominal p-value (p < 0.05) was considered as significant. This strategy for significance testing was chosen a priori. The rational for this choice is that many of the proteins are related to each other, and therefore Bonferroni-adjustment is too strict.

The proteins being validated to associate with 5 of the MetS components in the cross-sectional analysis were taken forward to the longitudinal analyses of MetS incidence during 5 years follow-up in the PIVUS study. In this part the subjects with MetS at baseline were excluded. Logistic regression analyses were carried out for the validated proteins vs incident MetS and these models were adjusted for sex and the life-style factors smoking, alcohol and energy intake, exercise habits, and education level. In this step, the limit of the p-value for significance was pre-specified as a Bonferroni-adjustment (p < 0.05/no. of tests). In secondary analyses, we also investigated associations with proteins that were associated with 4 MetS components in the replication step, and incident MetS.

In the Mendelian randomization (MR) part of the study, data from the SCALLOP CVD-1 project [17] were used to find SNPs suitable as instrumental variables for IL-1RA. Only SNP with p < 5*10–8 and being independent from each other were used as instrumental variables.

The independency of potential SNPs to be used as instruments for IL-1RA was previously evaluated in reference 17 by conditioning on the primary signal using conditional-joint analysis in GCTA (version 1.26.0) followed by filtering for MAF (0.01) and r^2^ (< 0.001) to ensure that secondary association signals identified were not driven by linkage disequilibrium (LD) with the primary signal.

Two cis-SNPs were found, which were evaluated to be causally related to MetS using a published genome-wide association study (GWAS) for MetS in UK biobank [10]. The causal estimates (Wald ratio) were calculated for each of those two SNPs, and those results were meta-analyzed using the inverse variance weighted method (IMW). P < 0.05 was considered significant in this meta-analysis of the Wald ratios. In addition, as an exploratory analysis, we evaluated if any SNPs in or close to the IL-1 alpha and beta genes (the ligands for the IL-1 receptor) were related to MetS in the GWAS of MetS in the UK biobank. The package MRbase in R (4.0.4) was used for LD-pruning of the SNPs identified in or close to the IL-1 alpha and beta genes.

STATA16.1 (Stata inc, College Station, TX, USA) was used for the statistical analysis (www.stata.com).

## Results

### Comparison of the two population-based samples

The ULSAM sample was older than the PIVUS sample and consisted of men only. The ULSAM sample also showed less subjects with an education > 12 years, less smokers, a higher proportion of subjects performing regular exercise, as well as higher proportions of the MetS criteria for triglycerides and blood pressure, but lower proportion of the waist circumference criteria when compared to the PIVUS sample. No differences with a p-value < 0.05 were seen for the proportion of MetS or the MetS criteria for glucose or HDL-cholesterol.

### Protein measurements

Of the 92 proteins included in the CVD-1 chip, 86 showed a call-rate above 75%. All but 8 showed a call-rate > 95% and 67 showed a call-rate of 100%. The six excluded proteins were: β-nerve growth factor, SIR2-like protein, interleukin-4 [IL-4], brain natriuretic peptide [BNP], nuclear factor-κB essential modulator and melusin [ITGB1BP2].

These 86 proteins were available in 996 subjects in the PIVUS study and in 785 subjects in the ULSAM study.

### The different MetS components vs proteomics in cross-sectional analysis

**Fig. 1:**
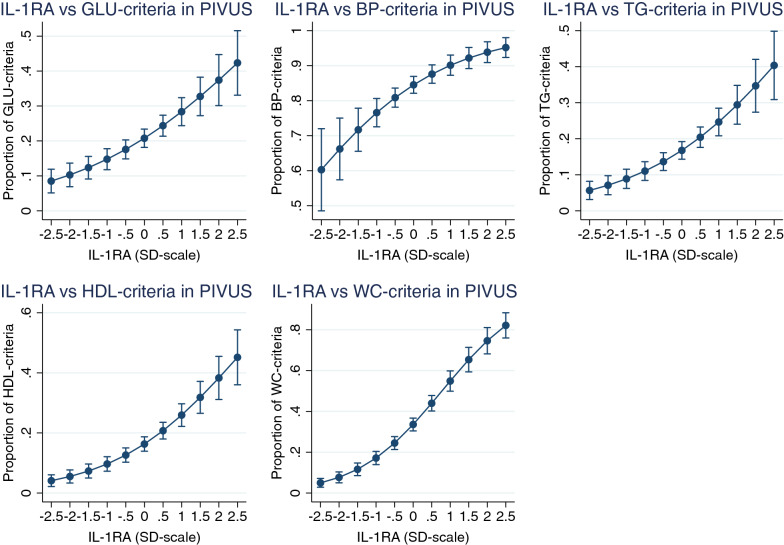
Relationships between Interleukin-1 receptor antagonist (IL-1RA) and the five components of the metabolic syndrome in PIVUS; glucose (GLU), blood pressure (BP), triglycerides (TG), HDL-cholesterol and waist circumference (WC). Logistic regression analysis was used to relate the proportion of these 5 components to IL-1RA. Graph is given with margins (and 95% CI) at each 0.5 SD, since IL-1RA is given at a SD-scale

Using logistic regression analysis, nine of the 86 proteins were related to all five components of the MetS in PIVUS following adjustment for life-style factors using FDR < 0.05 (Leptin, Fatty acid-binding protein 4 (FABP4), Tissue-type plasminogen activator (t-PA), Interleukin-1 receptor antagonist protein (IL-1RA, see Fig. 1), Hepatocyte growth factor (HGF), Tumor necrosis factor ligand superfamily member 14 (TNFSF14), Cathepsin D (CTSD), C–C motif chemokine 3 (CCL3), Chitinase-3-like protein 1 (CHI3L1)) (for details see Additional file 1: Table S1).

**Fig. 2:**
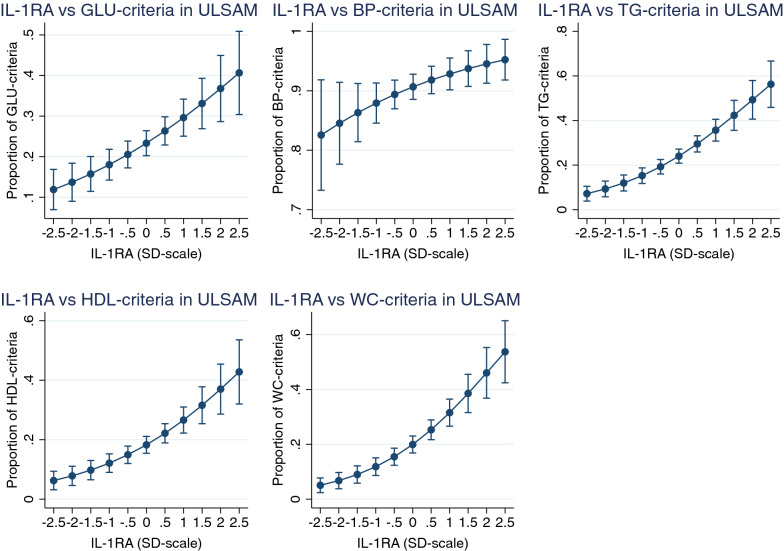
Relationships between Interleukin-1 receptor antagonist (IL-1RA) and the five components of the metabolic syndrome in ULSAM; glucose (GLU), blood pressure (BP), triglycerides (TG), HDL-cholesterol and waist circumference (WC). Logistic regression analysis was used to relate the proportion of these 5 components to IL-1RA. Graph is given with margins (and 95% CI) at each 0.5 SD, since IL-1RA is given at a SD-scale

Of those nine proteins, only IL-1RA was related to all five components of the MetS in ULSAM following adjustment for life-style factors using p < 0.05 when applying logistic regression analysis (see Fig. 2). Leptin, FABP4, t-PA, HGF, and CTSD was associated with all MetS components with the exception of blood pressure. See Table 2 and Additional file 1: Table S2 for details.

**Table 2:** Odds ratio (OR), standard error (SE), Wald z, *p*-values and 95% CI for the six proteins that in the PIVUS study were significantly associated with all five metabolic syndrome (MetS) components in PIVUS (false discovery rate at 5%) and with at least 4 of the 5 MetS components in the ULSAM study (at nominal p-values p < 0.05)

	OR	SE	z	*p*-value	95% CI
*Leptin*					
PIVUS					
Fasting glucose	1.34	.14	2.79	5.3e−03	1.09–1.66
Blood pressure	1.86	.22	5.15	2.7e−07	1.47–2.37
Triglycerides	1.51	.17	3.64	2.7e−04	1.21–1.89
HDL−cholesterol	1.58	.18	4.01	6.1e−05	1.26–1.99
Waist circumference	6.27	.90	12.72	4.7e−37	4.72–8.32
ULSAM					
Fasting glucose	1.74	.20	4.61	4.0e−06	1.37–2.20
Blood pressure	1.09	.18	0.54	5.9e−01	.78–1.52
Triglycerides	1.85	.21	5.24	1.6e−07	1.47–2.33
HDL-cholesterol	1.36	.16	2.59	9.6e−03	1.07–1.73
Waist circumference	5.50	1.09	8.56	1.1e−17	3.72–8.13
*t-PA*					
PIVUS					
Fasting glucose	1.22	.10	2.32	2.0e−02	1.03–1.46
Blood pressure	1.29	.11	2.79	5.3e−03	1.07–1.55
Triglycerides	1.74	.17	5.51	3.7e−08	1.43–2.13
HDL-cholesterol	1.46	.14	3.81	1.4e−04	1.20–1.77
Waist circumference	1.86	.15	7.30	2.8e−13	1.57–2.20
ULSAM					
Fasting glucose	2.28	.28	6.68	2.4e−11	1.79–2.90
Blood pressure	1.09	.15	0.65	5.2e−01	.83–1.43
Triglycerides	2.43	.30	7.05	1.8e−12	1.90–3.11
HDL-cholesterol	1.50	.17	3.52	4.4e−04	1.19–1.89
Waist circumference	2.12	.27	5.83	5.5e−09	1.64–2.73
*FABP4*					
PIVUS					
Fasting glucose	1.29	.12	2.78	5.5e−03	1.08–1.56
Blood pressure	1.40	.15	3.20	1.4e−03	1.14–1.73
Triglycerides	1.79	.18	5.61	2.0e−08	1.46–2.19
HDL-cholesterol	1.65	.17	4.72	2.3e−06	1.34–2.03
Waist circumference	3.02	.30	10.94	0.0001	2.47–3.68
ULSAM					
Fasting glucose	1.58	.16	4.42	9.7e−06	1.29–1.93
Blood pressure	1.20	.17	1.29	2.0e−01	.90–1.59
Triglycerides	1.94	.21	6.08	1.2e−09	1.57–2.41
HDL-cholesterol	1.36	.14	2.89	3.9e−03	1.10–1.68
Waist circumference	2.87	.39	7.79	6.5e−15	2.20–3.75
*IL-1RA*					
PIVUS					
Fasting glucose	1.45	.11	4.66	3.1e−06	1.24–1.71
Blood pressure	1.66	.18	4.54	5.5e−06	1.33–2.06
Triglycerides	1.56	.13	5.31	1.1e−07	1.32–1.84
HDL-cholesterol	1.85	.16	6.87	6.2e−12	1.55–2.21
Waist circumference	2.48	.22	10.20	2.0e−24	2.08–2.95
ULSAM					
Fasting glucose	1.39	.13	3.45	5.5e−04	1.15–1.68
Blood pressure	1.37	.20	2.09	3.7e−02	1.01–1.84
Triglycerides	1.80	.18	5.73	1.0e−08	1.47–2.20
HDL-cholesterol	1.52	.15	4.07	4.8e−05	1.24–1.87
Waist circumference	1.71	.18	5.03	4.9e−07	1.39–2.12
*HGF*					
PIVUS					
Fasting glucose	1.38	.11	3.87	1.1e−04	1.17–1.63
Blood pressure	1.26	.12	2.33	2.0e−02	1.03–1.54
Triglycerides	1.46	.12	4.32	1.6e−05	1.23–1.73
HDL-cholesterol	1.81	.17	6.29	3.3e−10	1.50–2.18
Waist circumference	1.93	.16	7.83	5.0e−15	1.64–2.28
ULSAM					
Fasting glucose	1.39	.13	3.34	8.3e−04	1.14–1.69
Blood pressure	1.05	.14	0.37	7.1e−01	.79–1.38
Triglycerides	1.46	.14	3.79	1.5e−04	1.20–1.78
HDL-cholesterol	1.30	.13	2.50	1.3e−02	1.05–1.60
Waist circumference	1.50	.16	3.81	1.4e−04	1.22–1.86
*CTSD*					
PIVUS					
Fasting glucose	1.63	.14	5.60	2.1e−08	1.37–1.93
Blood pressure	1.27	.12	2.49	1.3e−02	1.05–1.55
Triglycerides	1.82	.16	6.49	8.3e−11	1.52–2.19
HDL-cholesterol	1.73	.16	5.69	1.2e−08	1.43–2.09
Waist circumference	1.73	.14	6.75	1.5e−11	1.47–2.03
ULSAM					
Fasting glucose	1.92	.21	5.92	3.3e−09	1.55–2.39
Blood pressure	1.05	.14	0.37	7.1e−01	.80–1.38
Triglycerides	1.70	.18	5.00	5.8e−07	1.38–2.09
HDL-cholesterol	1.44	.16	3.29	1.0e−03	1.15–1.79
Waist circumference	1.56	.17	3.98	7.0e−05	1.25–1.94

OR, p-values, and 95% CI are given in both the PIVUS study and the ULSAM study for the different MetS criteria. t-PA: Tissue-type plasminogen activator; FABP4: Fatty acid-binding protein 4; IL-1RA: Interleukin-1 receptor antagonist protein; HGF: Hepatocyte growth factor; CTSD: Cathepsin D

### Longitudinal analysis of incident MetS in PIVUS

**Fig. 3:**
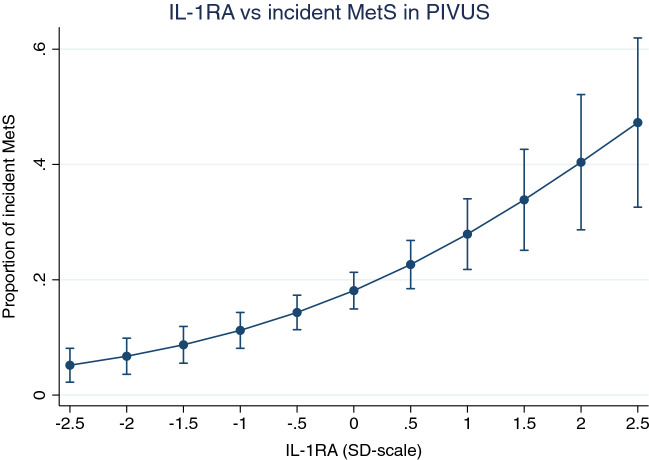
Relationships between Interleukin-1 receptor antagonist (IL-1RA) and incident metabolic syndrome (MetS) in PIVUS. Logistic regression analysis was used to relate the proportion of incident MetS to IL-1RA. Graph is given with margins (and 95% CI) at each 0.5 SD, since IL-1RA is given at a SD-scale

**Fig. 4:**
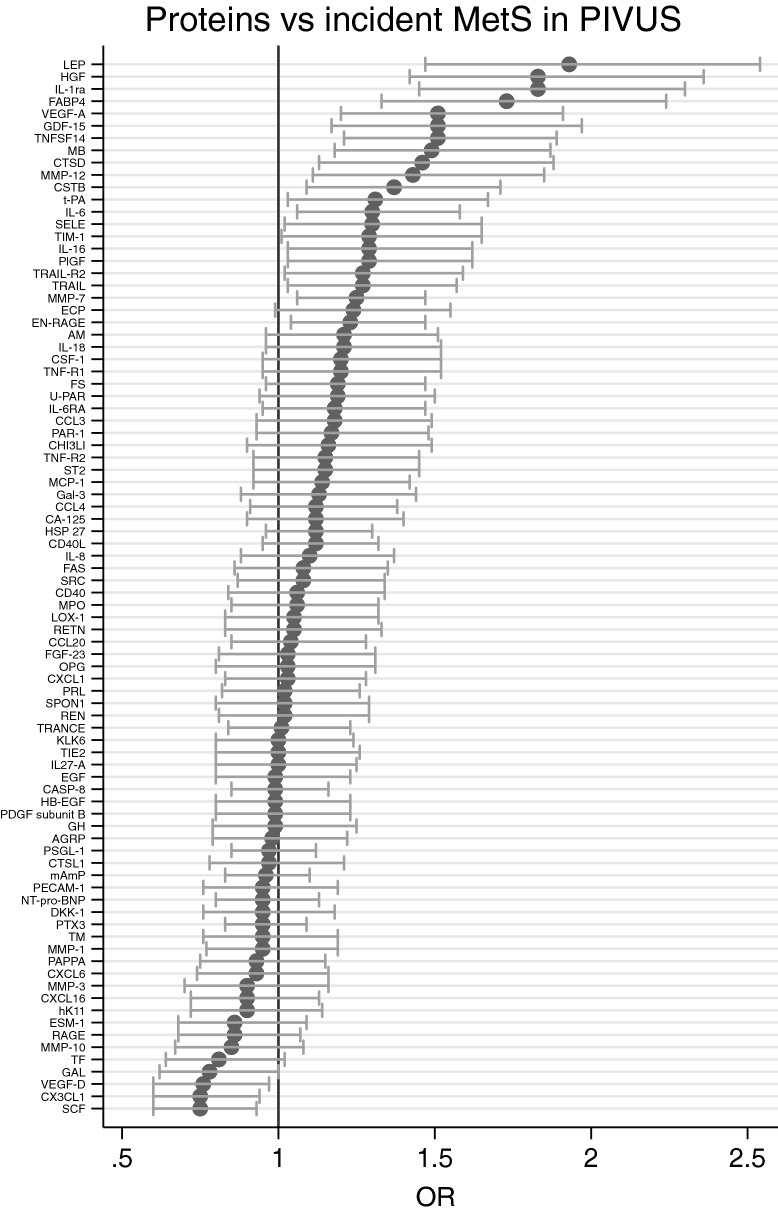
Relationships between the 86 evaluated proteins and incident metabolic syndrome (MetS) over 10 years follow-up in PIVUS. Logistic regression analysis was used for calculation of odds ratios (OR) and 95% CI. For abbreviations of the proteins, please see Additional file 1: Table S1

Using logistic regression analysis, IL-1RA was significantly related to incident MetS in the PIVUS study during 5 years follow-up (number of events = 109) when adjusted for smoking, exercise habits, education, and energy and alcohol intake (HR 1.76 for a 1 SD change (95% CI 1.38, 2.24), p = 4.3*10^–6^, see Fig. 3). For all 86 proteins vs incident MetS in PIVUS, please see Fig. 4.

When the five replicated proteins identified as being related to four of the different components of the MetS were evaluated in relation to incident MetS in the PIVUS study in a secondary analysis using logistic regression analysis, all but t-PA were associated with incident MetS following Bonferroni-adjustment for 5 tests (HR 1.93 (95% CI 1.46, 2.55) for HGF, HR 1.76 (1.38, 2.24) for IL-1RA, HR 1.88 (1.42, 2.48) for leptin, HR 1.76 (1.33, 2.32) for FABP4, HR 1.49 (1.14, 1.94) for CTSD, and HR 1.32 (1.02, 1.70), p = 0.034, for t-PA). Data for the proteins showing p < 0.05 in relation to incident MetS in PIVUS over 5 years are given in Table 3.

**Table 3:** Proteins significantly associated with incident metabolic syndrome (MetS, number of events = 109) in the PIVUS study during 5 years follow-up. OR, odds ratio

Protein	OR (95% CI)	p-value
Hepatocyte growth factor (HGF)	1.93 (1.46, 2.55)	3.23*10^–6^
Interleukin-1 receptor antagonist protein (IL-1RA)	1.76 (1.38, 2.24)	4.32*10^–6^
Leptin (LEP)	1.88 (1.42, 2.48)	.000011
Fatty acid-binding protein 4 (FABP4)	1.76 (1.33, 2.32)	.000065
Tumor necrosis factor ligand superfamily member 14 (TNFSF14)	1.52 (1.20, 1.92)	.00049
Vascular endothelial growth factor A (VEGF-A)	1.54 (1.2, 1.96)	.00055
Growth/differentiation factor 15 (GDF-15)	1.67 (1.24, 2.24)	.00061
Myoglobin (MB)	1.48 (1.16, 1.90)	.0017
Cathepsin D (CTSD)	1.49 (1.14, 1.94)	.0036
Stem cell factor (SCF)	0.71 (0.57, 0.90)	.0041
Matrix metalloproteinase-12 (MMP-12)	1.47 (1.12, 1.93)	.0060
Galanin peptides (GAL)	0.73 (0.56, 0.94)	.015
Cystatin-B (CSTB)	1.34 (1.06, 1.71)	.016
Placenta growth factor (PlGF)	1.34 (1.05, 1.71)	.019
Fractalkine (CX3CL1)	0.76 (0.60, 0.96)	.021
Interleukin-6 (IL-6)	1.27 (1.03, 1.57)	.022
Matrix metalloproteinase-7 (MMP-7)	1.22 (1.03, 1.45)	.022
Vascular endothelial growth factor D (VEGF-D)	0.75 (0.58, 0.97)	.029
Tissue factor (TF)	0.76 (0.59, 0.97)	.030
TNF-related apoptosis-inducing ligand receptor 2 (TRAIL-R2)	1.31 (1.03, 1.67)	.030
Tissue-type plasminogen activator (t-PA)	1.32 (1.02, 1.7)	.034
E-selectin (SELE)	1.3 (1.01, 1.67)	.039

### Mendelian randomization of IL-1RA vs MetS

Two cis-SNPs (rs12990810 and rs6734238) were identified to be GWAS-significantly related to IL-1RA levels in the SCALLOP consortium [17]. However, when using those as instrumental variables in a Mendelian randomization analysis using the GWAS of MetS in the UK biobank applying a meta-analysis of the two Wald ratios, no evidence for a causal effect of IL-1RA on MetS was found (beta 0.009, 95% CI − 0.052 to 0.070, p = 0.77).

In an exploratory analysis, we further evaluated if the 150 SNPs in and near the IL1-alpha and IL1-beta genes (2:113531491–113542167 and 2:113587327–113594480, respectively, using GRCh37/hg19) were related to MetS using the GWAS of MetS in the UK biobank. Following LD-pruning, these 150 SNPs represent 6 independent loci (rs112536764, rs114021768, rs115781547, rs116681462, rs138451217 and rs41294736). The top locus of the 150 evaluated SNPs (rs3136558) showed p = 0.0012, being far from GWAS-significant (see Additional file 1: Table S3).

## Discussion

The present study discovered and validated one protein to be related to all five components of the MetS (IL-1RA). IL-1RA was also related to incident MetS over 5 years of follow-up in the longitudinal evaluation, but a Mendelian randomization did not find support for a casual association between circulating levels of this protein and the MetS.

### Comparison with the literature

We have previously reported an analysis of the proteomic profile of different MetS components using 249 proteins in another community-based sample (the EpiHealth study, n = 2444) [12]. In that study, twenty proteins were related to all five of the MetS components following Bonferroni-adjustment. IL-1RA, the most interesting protein in the present study, was amongst the top ranked proteins in terms of relationships with all MetS components also in the EpiHealth study. Of the other proteins being related to five or 4 components in both PIVUS and ULSAM, Leptin, FABP4, t-PA, HGF, and CTSD, all but HGF were related to all 5 MetS components in the EpiHealth cohort. The EpiHealth study had the advantage to be almost 2.5 time larger than the PIVUS study used for discovery, and thereby the power was greater. However, the EpiHealth study was only cross-sectional in its character, did not include any validation step in a separate sample, nor were Mendelian randomization analyses performed to assess causality. Thus, the novelty and strength of the present study is the validation in an independent sample, the extension of the cross-sectional analyses to a longitudinal setting, and the use of Mendelian randomization to address causality.

IL-1RA is an endogenous antagonist to IL-1-alpha and beta, and is elevated in proportion to the activation of IL-1 [18]. Since this protein is easier to detect in plasma than the agonists, this is a commonly used as a marker for IL-1-pathway activation. The fact that IL-1RA was related to all five of the components of MetS, including the blood pressure criteria, made this protein an interesting candidate for further exploration. The fact that is was related also to incident MetS in the longitudinal analysis might indicate that this protein, or the IL-1 pathway, could be of pathogenetic importance for the clustering of risk factors, but it could not excluded that high IL-1RA levels are a consequence of the syndrome rather than causing MetS. This latter explanation is probably the most likely, since our two-sample MR analysis did not show any causal effect of IL-1RA on MetS, and the recently published SCALLOP analysis of proteins on the CVD-1 chip showed that genetic instruments for BMI, as well as for percent body fat, were causally related to IL-1RA levels [17]. The link to body fat is further supported by a previous study reporting increased expression of IL-1-beta and IL-1RA in adipose tissue in obese as compared to lean subjects [19], and weight loss has been linked to reduced adipose tissue expression of these cytokines [20]. It has also been shown that visceral adipose tissue (VAT) releases more IL-1-beta than subcutaneous adipose tissue (SAT) [21]. Since excess visceral adipose tissue is particularly linked to MetS [22], this might explain the relationship vs MetS also revealed in the longitudinal analysis.

Another 5 proteins were related to four of the five MetS components; Leptin, FABP4, t-PA, HGF, and CTSD. For all of those, no certain link could be established vs the blood pressure component. All of those, except t-PA, were associated also with incident MetS. Interestingly, IL-1RA and the other five proteins found to be related to five or four of the MetS components were amongst the proteins previously validated to be linked to insulin resistance (HOMA-index) in the ULSAM and PIVUS studies [23]. That was an expected finding since HOMA-index was closely related to MetS in both cohorts (p < 0.0001 in both samples).

We could generally see that the blood pressure component is less likely to be significantly related to proteins than other MetS components, especially in the ULSAM cohort. This was also seen in a previous study [12]. There could be different reasons why blood pressure is the MetS component that is related to the lowest number of proteins. First, blood pressure is a rather unstable measurement with a lot of variation during the day and between days. A measurement of HDL or waist circumference is more stable from day to day. Second, although related to obesity and insulin resistance, blood pressure is a hemodynamic variable, while the other four components more directly reflect metabolism. Third, the prevalence of the blood pressure criteria was very high in both PIVUS and especially ULSAM (90%), so the power to detect significant relationships vs this MetS component is rather low compared to the others. No significant interactions were seen between the proteins of interest and sex regarding the blood pressure component in PIVUS. Thus, the fact that the validation step only included men does not seem to explain the low number of proteins being related to blood pressure in ULSAM.

Only one of the nine proteins identified in the discovery step were replicated in the validation step. This could be due to different reasons. First, some of the discovery findings could be chance findings. Two, the two cohorts differ both in time of collection of data, as well as in age. Third, although the same methods were used for collection of data to be used to define MetS components, drifts in laboratory methods might have occurred over time. Fourth, although, we did not find any sex-interactions of interest in the PIVUS discovery stage, the fact that ULSAM consists of men only might still have an impact. Fifth, the ULSAM sample is smaller and therefore have less power to detect significant relationships. Thus, several factors may cause that many proteins were not replicated. However, it is still our belief that it is better to only report replicated findings to avoid false positive results in the scientific literature.

As could be seen in Table 1, the two samples differ not only in age and sex-distribution, but also in other characteristics, such as smoking, exercise habits and education. However, in order to be regarded as a robust reproduced result, such as IL-1RA, the result has to be validated in samples being not identical to the discovery sample.

The strength of the present study is that we could evaluate a large number of proteins in relation to the MetS components using independent cohorts and both cross-sectional and longitudinal data. A limitation is that the proteins included in the present proteomics assay were selected for their involvement in atherosclerosis and cardiovascular disease, rather than insulin resistance or other dimensions of metabolic disturbances. We also acknowledge the fact that the study was performed in homogenous populations of elderly Swedes, thus our findings require replication in other age and ethnic groups.

In conclusion, IL-1RA was related to all five components of the MetS in a cross-sectional analysis in two independent samples, as well as to incident MetS in a longitudinal analysis, but was not causally related to MetS as evaluated by Mendelian randomization analyses.

## Supplementary Information

**Additional file 1: Table S1.** Relationships between the 86 evaluated proteins and the five components of the metabolic syndrome (MetS) in the cross-sectional evaluation in PIVUS. Odds ratio (OR) and 95% CI are geiven together with -pvalue for each ot the five components. GLU = glucose, BP = blood pressure, HDL = HDL-cholesterol, TG = triglycerides, WC = waist circumference. A p-value of 0 denotes a p-value < 10^–7^. The short names of the proteins are used in Fig. 4. **Table S2.** Relationships between the 86 evaluated proteins and the five components of the metabolic syndrome (MetS) in the cross-sectional evaluation in ULSAM. Odds ratio (OR) and 95% CI are given together with p-value for each of the five components. GLU = glucose, BP = blood pressure, HDL = HDL-cholesterol, TG = triglycerides, WC = waist circumference. A p-value of 0 denotes a p-value < 10^–7^. The short names of the proteins are used in Fig. 4. **Table S3.** Relationships between SNPs in the region of the genes for IL-1 alpha and beta on chromosome 2 and the metabolic syndrome (MetS) in UK biobank (Lind L. *Metab Syndr Relat Disord*. 2019;17:505-11).

## Data Availability

Due to Swedish law and the Ethical Committee permission, health data at the individual level cannot be made available online as an open source for the public. Data are however available by a request sent to the corresponding author.

## Abbreviations

CCL3: C–C motif chemokine 3
CHI3L1: Chitinase-3-like protein 1
CTSD: Cathepsin D
FABP4: Fatty acid-binding protein 4
FDR: False discovery rate
GWAS: Genome-wide association study
HGF: Hepatocyte growth factor
IL-1RA: Interleukin-1 receptor antagonist protein
MetS: Metabolic syndrome
MR: Mendelian randomization
PEA: Proximity extension assay
PIVUS: The Prospective Investigation of the Vasculature in Uppsala Seniors
t-PA: Tissue-type plasminogen activator
TNFSF14: Tumor necrosis factor ligand superfamily member 14
ULSAM: The Uppsala Longitudinal Study of Adult Men
